# Comparisons in Screen-Time Behaviours among Adolescents with and without Long-Term Illnesses or Disabilities: Results from 2013/14 HBSC Study

**DOI:** 10.3390/ijerph15102276

**Published:** 2018-10-17

**Authors:** Kwok W. Ng, Lilly Augustine, Jo Inchley

**Affiliations:** 1 Health Research Institute, University of Limerick, Limerick V94 T9PX, Ireland; 2 Centre of Physical Activity and Health Research, University of Limerick, Limerick V94 T9PX, Ireland; 3 Department of Physical Education and Sport Sciences, University of Limerick, Limerick V94 T9PX, Ireland; 4 CHILD, School of Education and Communication, Jönköping University, 55 111 Jönköping, Sweden; lilly.augustine@ju.se; 5 School of Medicine, University of St. Andrews, St. Andrews KY16 9TF, UK; jci2@st-andrews.ac.uk

**Keywords:** sedentary behaviours, screen time, children, HBSC, disability

## Abstract

Reducing sedentary behaviours can help prevent non-communicable diseases, particularly among young adolescents with long term illnesses or disabilities (LTID). Much of young people’s voluntary sedentary time is related to screen-time behaviours (STBs) such as TV viewing, playing computer games, and using the computer for other activities. Although public health data on adolescents’ STB is growing, information about adolescents with LTID is currently lacking in a European context. The purpose of this study is to compare time on STBs between adolescents with and without LTID in European Countries through the HBSC 2013/14 study. Young adolescents (n = 61,329; boys 47.8%) from 15 European countries reported the time spent on TV viewing, playing computer games, and using the computer for other purposes on weekdays and the weekend. STBs were dichotomised based on international recommendations of less than 2 h per day, and Chi-square tests of independence were performed to investigate differences. STB time was combined to produce a sum score as dependent variable in multiple analysis of covariance with age and family affluence as covariates. There were statistically significant differences in computer gaming among boys and other computer use among girls for both weekdays and weekends, whereby adolescents with LTID reported higher use. In addition, both boys and girls with LTID spent more time on STBs than their same sex peers without LTID (Boys, F = 28.17, *p* < 0.001; Girls, F = 9.60, *p* = 0.002). The results of this study indicate a need for preventive strategies to address high levels of STB among young adolescents with LTID and reduce the risk of poor health outcomes associated with higher levels of sedentary behaviour.

## 1. Introduction

Reducing sedentary behaviours is one of the main goals in preventing non-communicable diseases. However, rapid changes in availability of digital technologies has facilitated sedentary behaviours [1]. Engagement in screen-time behaviours (STB), such as watching the TV, playing computer games, or using computers for other purposes, such as social media or surfing the internet, is common among adolescents and contributes to overall sedentary time [2,3,4]. Prolonged STB can have negative effects on physical and psychological health [5,6]. There are also longitudinal associations between STB and overweight and obesity [7].

Evidence of associations between sedentary behaviour and poor health outcomes, such as obesity, low fitness and low self-esteem [8], has led to recommended limits of less than two hours per day of time spent on STB [9,10]. Despite these recommendations, there is evidence to suggest that a high proportion of adolescents do not keep to these limits, and this has been increasing over time [11]. School-aged children have the opportunity to spend more time in STB during the weekends, therefore it is important to examine the behaviours during the week and separately on the weekends [4]. Moreover, boys tend to report more time on STBs than girls, especially in computer games [12].

The World Health Organisation defines adolescence as 10–19 years, although there have recently been calls to extend this age range through to 25 years old [13]. Major physical and social transitions take place during the early adolescent years, including the transition from primary to secondary level education, and critical biopsychosocial changes associated with puberty [14]. This is also the stage at which substantial declines in physical activity are typically observed and new lifestyle habits are established. Young adolescents with long-term illnesses or disabilities (LTID) are at risk of developing further health conditions through sedentary lifestyles [15]. Young adolescents with LTID are often excluded from different types of exercise programs, both in school and after-school, due to inaccessibility making them more likely to engage in sedentary activities such as watching TV [16]. In the school setting, young adolescents with LTID may be placed in environments with more sedentary activities rather than active ones. Young adolescents with LTID may have more school absenteeism due to their condition as well as other exclusions from school life, such as physical education (PE) class and field trips [17]. This type of exclusion from activities can be harmful to the child’s social and emotional development because less time is spent with friends [18]. Taking part in STB, particularly computer-related activities, may offer an alternative for social interaction, especially for young adolescents with disabilities who might feel lonelier and more isolated than others [19]. However, excessive use may lead to poor mental health through internet addiction [20]. Monitoring prevalence and enabling group comparisons, such as between people with and without LTID, are needed to help inform development of more targeted interventions. 

The research on STB among children with LTID is limited. There have been some small investigations of overall sedentary time measured using accelerometers, whereby children with LTID generally spend more time being sedentary than their peers without LTID [21]. In particular, adolescents with cerebral palsy were more inclined to spend more time playing electronic games [22]. However, some studies suggest the differences between children with and without autism spectrum disorder were not statistically significant [23]. This confirmed a previous study whereby differences were not statistically significant after controlling for secondary health conditions and medication use [24]. On the other hand, there are reports of adolescents with other types of disabilities than autism spectrum disorder who spend less time on specific STBs, such as TV viewing time [25]. As such, it remains unclear whether there are differences among adolescents with LTID and if patterns of behaviour vary between countries. 

From the perspective of public health, data that are disaggregated by disability are needed for monitoring purposes across different contexts to inform appropriate policies which aim to reduce STBs. Therefore, in this study, we use cross sectional data from various countries across Europe and compare the differences in STB among young adolescents with and without LTID. Sedentariness is a risk factor for future chronic diseases, therefore reducing adolescents’ sedentary behaviour is essential for promoting good health now and into adulthood. Recommendations have been made of a maximum of two hours of screen time per day to reduce risk. Screen behaviour today can often include simultaneous use of different devices [26], therefore a higher cut off might be needed. By investigating patterns of STB among adolescents in 15 countries, disaggregated for LTID, we aim to investigate whether prevalence of STB varies by disability status and country of residence. 

## 2. Materials and Methods 

Data were collected from the World Health Organization cross-national collaborative Health Behaviour in School-aged Children (HBSC) study in Europe, North America and Israel. Young adolescents aged 11-, 13-, and 15-years old are included in the school-based survey, following a well-established international protocol for data collection in each participating country. The primary sampling units are the schools and classes and ethical approval for the study and consent for participation are carried out at the national level. According to the protocol, the survey consists of three types of questions: (i) mandatory international items; (ii) optional international items; and (iii) national items. All participating countries are requested to carry out data collection with the mandatory set of items. For this study, these items include the study demographics as well as items on screen time. Participating countries can choose if they would like to include optional items. The items in this study that fall under this category measure survey participant’s status with regard to LTID (yes or no). In accordance with the international study protocol, the language of the national questionnaires was checked through a back-translation protocol prior to data collection to ensure cross-national comparability. This process included checking for terminology and wording of the items as well as the response options. 

### 2.1. Sample

From each country, a nationally representative sample was created through a randomized cluster sampling procedure, with the cluster set at the school level. A total of 61,329 young adolescents (Boys 47.8%, Girls 52.2%; 11y 31.1%, 13y 34.7%, 15y 34.2%) provided full responses to all STB variables, LTID status and the covariates that were included. The survey was carried out in the classroom and was administered by the teachers assigned by the school who were given instructions in how to administer the survey. All participation was voluntary, anonymous and the right to withdraw at any time was possible. The administrators were not permitted to look at the results of the survey at the individual level to ensure confidentiality. Response rates were over 70% at the international study level and ranged between 40% and 92% at the national level. Many non-responses were reported to be absentees. The procedures for the survey can be downloaded from http://www.hbsc.org/methods/index.html.

### 2.2. Survey Items

All items related to STBs had the same response options whereby young adolescents were asked to select one option for weekdays and one option for weekends. Response options ranged from “none at all” to “About 7 or more hours a day”. Only in Slovakia, data on the weekend was not available. To measure the time spent watching TV during leisure time, participants were asked; “How many hours a day, in your free time, do you usually spend watching TV, videos (including YouTube or similar services), DVDs, and other entertainment on a screen?” Playing computer games was measured using the following question; “How many hours a day, in your free time, do you usually spend playing games on a computer, games console, tablet (like iPad), smartphone or other electronic device (not including moving or fitness games)?” Use of a computer for other purposes was measured using the following question; “How many hours a day, in your free time, do you usually spend using electronic devices such computers, tablets (like iPad) or smart phones for other purposes, for example, homework, emailing, tweeting, facebook, chatting, surfing the internet?”

These questions have been used repeatedly in the HBSC survey as indicators of STB. In 2001/2 study, a test-retest study over seven days took place in Belgium. The authors of that study reported strong agreement for boys (ICC = 0.76) and girls (ICC = 0.81) [27]. The stability of the items were tested again from the 2009/10 HBSC cycle across adolescents in China, where there was moderate agreement for computer games (weekdays, ICC = 0.54; weekends, ICC = 0.69) and lower agreement for using computer for other purposes (weekdays, ICC = 0.33; weekends, ICC = 0.50) [28]. The content validity of instruments that measure STB is considered difficult to assess [29]. However, using self-reported TV diaries and questionnaires, a previous study found that convergent validity was stronger among girls (Boys, ICC = 0.36; Girls, ICC = 0.54) [27]. 

Each of the six STB variables were dichotomized in order to compare the proportion of young adolescents who spent less than 2 h per day versus those who spends more than 2 h per day, thus corresponding to the international recommendations for STBs. In addition, a summed score of all the STB during the weekdays and the weekend were calculated. A cut off of 3 h per day was used to allow for time spent reporting multiple ST use, and to keep the results comparable to a recent international comparison study by Hoare and colleagues [12]. Subsequently, the summed score for all STBs combined was entered into the multivariate statistical analyses.

### 2.3. Covariates

The young adolescents were asked to report their gender as either boy or girl. At the time of the survey no other options were available. Prior literature suggests there are large differences in STB between boys and girls [11], hence the analyses were stratified by gender. Also, the young adolescents reported the month and year of birth. Their age was then calculated based on the time of completion of the survey. For the purposes of reporting age categories, they were grouped as 11-, 13- and 15-year-olds after rounding to the nearest age group. Relative wealth was measured through the Family Affluence Scale (FAS III) as a child-friendly indicator of socioeconomic status [30]. In all countries except for Armenia, the six item FAS III was used. In Armenia, the four item FAS II (number of computers, car ownership, family holidays in the past year, and having one’s own bedroom) was used. FAS III is updated from FAS II to reflect the changes in household possessions and is the same as FAS II, but with two additional items (dishwasher in household, number of bathrooms in household) [31]. All items were summed up, then a relative ridit score was created through a rankit command in IBM SPSS Statistics for Windows, Version 24.0. Quintiles were created and the lowest 20% represented low FAS, the middle 60% represented medium FAS, and the highest 20% represented high FAS.

### 2.4. Data Analyses

All analyses were carried out on IBM SPSS 24.0. Descriptive data were presented as proportion of young adolescents who spent two or more hours watching TV, playing computer games, or use of computer for other purposes for the total sample and for each country. Chi-square tests of independence were used for determining statistical differences based on, having LTID or not, and spending two hours or more or not, for each STB. Multivariate analyses were carried out at the individual level. Means and standard deviations of the summed STB were analysed with *t*-tests to investigate gender differences at the country level and for the pooled population. Repeated univariate analysis of covariance was undertaken with summed hours per day for week and weekend as the dependent variable, LTID status as the independent variable, controlling for age and family affluence. The level of statistical significance was 0.05. Results are presented separately for boys and girls and for weekdays and weekends. Data for this study can be made available by request through the HBSC data bank in Bergen, Norway (http://www.uib.no/en/hbscdata). 

## 3. Results

### 3.1. Watching TV by Countries

Prevalence of TV watching was higher at the weekend; approximately three-fifths of young adolescents spent two hours or more per day watching TV during the week and four-fifths did so during the weekend (Table 1). For boys only one significant difference was found: In Sweden, more boys with LTID watched more TV during weekdays than boys without (*p* = 0.002), no difference on weekends were found. In Sweden, fewer boys with LTID than without LTID (*p* = 0.002) reported watching TV on weekdays. In all other countries, there were no significant differences in watching TV on weekdays or at the weekend.

There were few significant differences regarding girls. Three countries indicated that more girls with LTID watched TV for two hours or more per day during weekdays (England: 66% vs. 60%; Ireland: 55% vs. 49%; and Sweden: 66% vs. 61%). On weekends more girls with LTID in the former Yugoslav Republic of Macedonia (86% vs. 77%) watched TV more than the recommended limits of two hours. 

### 3.2. Boys Computer Gaming and Use

Almost half of boys spent less than 2 h playing computer games or used the computer during the weekdays (Table 2). Boys with LTID were more sedentary through computer games and use of computers for other purposes during the weekdays (*p* < 0.001) and weekends (*p* = 0.011) than boys without LTID. More boys with LTID in Finland and in Sweden spent two hours or more per day playing computer games during the weekdays. After pooling the population data together (In Table 2, see row for ‘All’), more boys with LTID (*p* = 0.008) reported to use the computer use for other purposes than games for two hours or more per day during the weekdays. In Poland, there was a statistically significant difference (*p* = 0.005) in the proportion of boys with and without LTID who used computers for other purposes than gaming for two hours or more per day during weekdays. The differences in other countries were not statistically significant. 

Less than three quarter of boys played computer games for two hours or more per day during weekends, whereas three out of every five boys used computers for other purposes for two hours or more per day during the weekends. More boys with LTID (74.4% vs. 72.6%, *p* = 0.011) were playing computer games for two hours or more per day during the weekend than boys without LTID. More specifically, more boys with LTID in Hungary, Poland, and Wales spent two hours or more playing computer games on the weekend than boys without LTID. There were similar differences in Hungary and Poland in the use of computers for other purposes during weekends. However, the differences were not statistically significant across the pooled data or in other countries. 

### 3.3. Girls Computer Use and Gaming

During weekdays, less than one third of girls stayed within the recommended time limit regarding playing computer games (see Table 3). In England, the former Yugoslav Republic of Macedonia, and Sweden, girls with LTID were more sedentary than girls without LTID, by reporting to have played computer games for two hours or more per day during the week. Moreover, during the weekends, more girls with LTID in England and in Sweden played computer games for two hours or more per day than girls without LTID. Differences in other countries as well as the pooled sample were not statistically significant.

During weekdays more than half the girls reported using computers for other purposes than gaming for two hours or more per day, however during weekends almost two-thirds exceeded the two hour recommendation and girls with LTID were more sedentary. More girls with LTID reported two hours or more per day of computer use during the weekdays (*p* < 0.001) and the weekends (*p* = 0.001) than girls without LTID. Prevalence was higher among girls with LTID in Armenia, England, Finland, the former Yugoslav Republic of Macedonia, and Sweden on weekdays. Similar differences between girls with and without LTID were reported in England and Sweden for the weekend. Differences in other countries were not statistically significant. 

### 3.4. Sum Screen Time by Countries

The summed time of STB was greater among boys (mean = 6.8 h, SD = 4.8) than girls (mean = 6.0 h, SD = 4.4) on weekdays. During the weekend, boys (mean = 9.3 h, SD = 5.5) reported over one hour more than girls (mean = 8.1 h, SD = 5.1) of summed STB time (Table A1). The gender difference was statistically significant at the pooled population level for both weekdays (*p* < 0.001) and weekends (*p* < 0.001) and for each country, apart from STB during the weekdays in England (*p* = 0.743) and in Ireland for STB during the weekdays (*p* = 0.083) and weekends (*p* = 0.647). 

**Figure 1:**
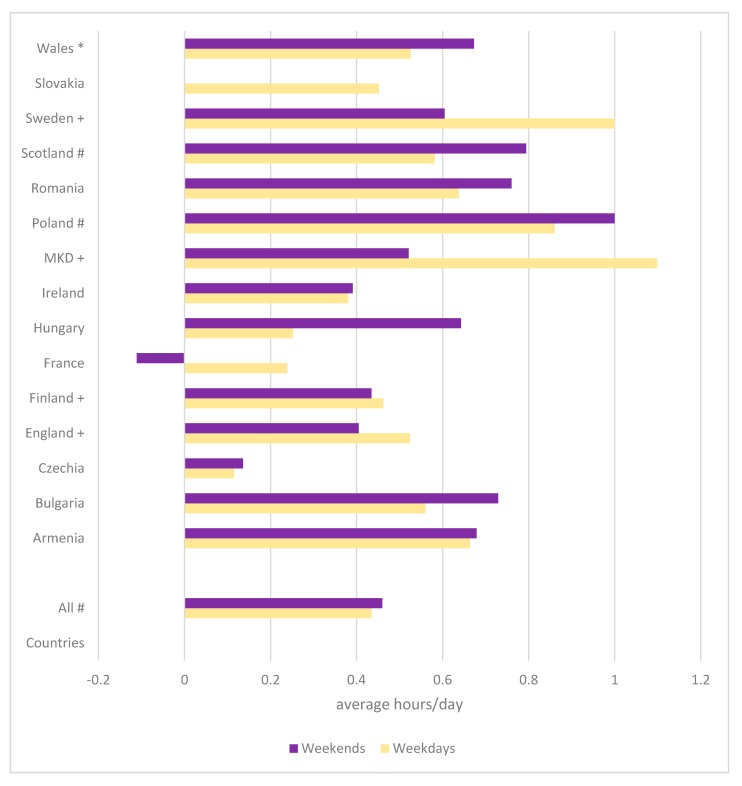
Difference in mean hours/day of screen time between Boys with and without LTID; + *p* < 0.05 for weekdays, * *p* < 0.05 for weekends, # *p* < 0.05 for weeks and weekends. MKD, the former Yugoslav Republic of Macedonia.

After adjusting for age and FAS, in the pooled population, there were statistically significant differences in reported total time STBs between boys with and without LTID during weekdays (F = 35.417, *p* < 0.001) and weekends (F = 28.170, *p* < 0.001), whereby boys with LTID spent more time on STBs than boys without LTID (Figure 1). At the country level, statistically significant differences were observed in six countries (England; F = 4.686, *p* = 0.031; Finland; F = 4.569, *p* = 0.033; the former Yugoslav Republic of Macedonia; F = 4.678, *p* = 0.031; Poland; F = 10.893, *p* = 0.001; Scotland; F = 4.969, *p* = 0.026; Sweden; F = 23.775, *p* < 0.001). Similarly, on the weekends, these differences were significant in four countries (Poland; F = 10.819, *p* = 0.001; Scotland; F = 7.758, *p* = 0.005; Sweden; F = 6.296, *p* = 0.012; Wales; F = 4.156, *p* = 0.042).

**Figure 2:**
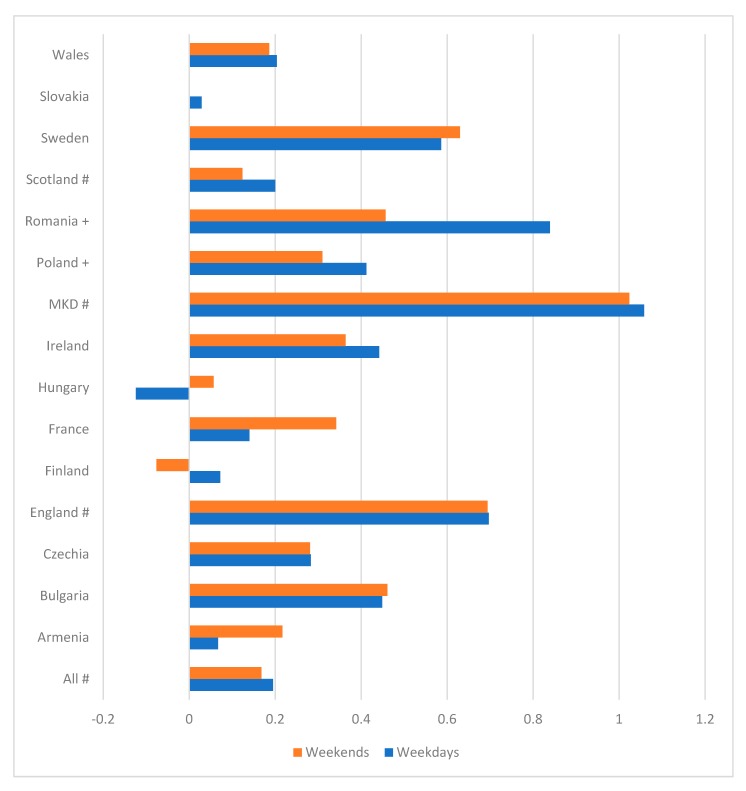
Difference in mean hours/day of screen time between Girls with and without LTID; + *p* < 0.05 for weekdays; *p* < 0.05 for weekends; # *p* < 0.05 for weeks and weekends; MKD, the former Yugoslav Republic of Macedonia.

On average, across all countries, girls with LTID reported more total time on STBs than those without LTID on weekdays (F = 9.599, *p* = 0.002) and weekends (F = 0.4894, *p* = 0.027) (Figure 2). At country level, these differences were statistically significant for weekday STB in five countries (England; F = 8.230, *p* = 0.004; the former Yugoslav Republic of Macedonia; F = 5.371, *p* = 0.021; Poland; F = 4.069, *p* = 0.044; Romania; F = 4.311, *p* = 0.038; Sweden, F = 12.727, *p* < 0.001). For the total time on STBs during the weekend, there were statistically significant differences in three countries (England (F = 6.719, *p* = 0.010), the former Yugoslav Republic of Macedonia (F = 3.891, *p* = 0.049), and Sweden (F = 10.448, *p* = 0.001)).

## 4. Discussion

In this study, screen time behaviour, measured by self-reported time spent on TV, computer games, and other uses of computers, was compared between young adolescents with and without LTID in 15 European countries. For each behaviour, boys reported, on average 1.5 h more screen time than girls during the week and 1.3 h during the weekend. There were no significant differences in the proportion of adolescents who met the recommendations of less than 2 h per day in TV viewing, but use of a computer for playing games or other purposes on weekdays was significantly higher among adolescents with LTID suggesting that these young people spend more time being sedentary than their peers without LTID. These differences were not consistent across all countries, indicating cultural variations in social norms for young people with LTID across Europe.

The amount of time spent on STBs is only part of the overall sedentary time that adolescents experience. The amount of composite time was distributed almost evenly, although TV viewing was the most common activity and computer gaming was the least common overall, for both weekdays and weekends. With the increasing pervasiveness of digital technologies in young people’s lives and the greater role of computers in education and extra-curriculum activities [32], it may be challenging to increase the proportion of young adolescents who adhere to the recommendations. Through replacement of sedentary time with light physical activity, there are immediate and long term health benefits [33,34]. Our results suggest that there is a difference in computer use between young people with and without LTID during the weekdays and weekend. Adolescents with LTID may feel they require more time on studies and get assistance through online resources [19]. However, they may also feel excluded from participating with other peers [22,35], due to inaccessibility or decreasing social participation [36] and therefore be more likely to use their leisure time on screen based activities. Online communication may also provide important opportunities for social interaction among young people who may be more socially isolated. Given the well-established health benefits of physical activity and the risks associated with sedentary behaviour, higher frequency of STB among adolescents with LTID is of concern for both current and future health outcomes. Some peer-led activities have been found to be useful in increasing physical activity levels among adolescents [37], suggesting that it may also be possible to use similar strategies to reduce sedentary behaviour and screen time in particular. Further research is required to identify effective prevention and intervention approaches. 

Many other comparison studies or reviews often do not report data based on adolescents with LTID. For example, an international trend study reported the changes in STBs among young adolescents [11] and there was no reporting on whether there were measures of LTID to know if the adolescents were included or not. Making data available to disaggregate by LTID is urgently needed for monitoring purposes as per the sustainable development goals [38]. Moreover, countries that have ratified the United Nations Convention on the Rights of Persons with Disabilities are also aware that collection of data by disaggregation is compliant to article 31 [39]. In our study, we have attempted to report the results after disaggregation by disability to help inform future studies in STBs.

Another study [12] that compared STBs of adolescents across countries explicitly identified individuals with LTID as an exclusion criteria in their searches, but the authors did not state a reason for it. One possible reason could be that studies involving individuals with LTIDs tend to have a smaller sample size. The prevalence of adolescents with LTID in this study was approximately 19% and has been reported elsewhere [40]. Therefore, we were able to carry out such analyses. We could not find statistically significant differences in TV viewing behaviours, therefore we suspect it is unnecessary to have an exclusion criteria for future reviews. Rather, we would encourage the use of disaggregation by LTID where possible, as we carried out in this study. This would give us more insights into addressing the sedentary behaviours across various population groups.

The results of this study have some limitations that need to be addressed when considering the findings. Our measures of STB were self-reported and there may have been some error in the reporting because in recent times it is possible to use the TV and computer simultaneously, leading to over reporting the amount of sedentary behaviour [26]. Measures of self-reported LTID may have different internal validity across the countries included. However, the HBSC data used in the study are based on a common protocol allowing robust comparability of findings across different countries.

## 5. Conclusions

In summary, the adolescents with LTID in our study reported spending more time on STBs during weekdays and weekends than same sex peers without LTID. Although measuring time spent viewing TV, playing computer games, and using the computer for other purposes does not capture all means of being sedentary, they are still behaviours that remain at the forefront in methodologies in pan-European surveillance [41]. There is a global need to address reducing sedentary time throughout the waking hours. Separate intervention strategies may be needed when targeting adolescents with LTID.

## Figures and Tables

**Table 1:** Proportion (0–1) of boys and girls who spend two hours or more per day watching TV during the week and the weekend.

	Boys								Girls							
	TV Weekday					TV Weekend			TV Weekday					TV Weekend		
	No LTID		LTID			No LTID	LTID		No LTID		LTID			No LTID	LTID	
Country	N	>2 h	N	>2 h	*p*	>2 h	>2 h	*p*	N	>2 h	N	>2 h	*p*	>2 h	>2 h	*p*
All	22480	0.62	5352	0.63	0.24	0.79	0.79	0.55	24967	0.59	5998	0.6	0.15	0.77	0.78	0.50
Armenia	857	0.60	76	0.60	0.89	0.77	0.72	0.34	1233	0.57	67	0.55	0.76	0.75	0.77	0.79
Bulgaria	1952	0.69	197	0.72	0.47	0.74	0.72	0.44	1888	0.72	245	0.75	0.38	0.78	0.8	0.42
Czechia	1714	0.63	612	0.61	0.39	0.77	0.79	0.17	1846	0.54	729	0.58	0.12	0.7	0.72	0.31
England	1555	0.60	482	0.62	0.39	0.8	0.8	0.77	**1543**	**0.6**	**488**	**0.66**	**0.03**	0.81	0.8	0.49
Finland	1018	0.64	428	0.61	0.30	0.81	0.82	0.95	1141	0.56	527	0.53	0.20	0.8	0.78	0.34
France	1930	0.57	513	0.59	0.43	0.79	0.77	0.34	1991	0.52	517	0.54	0.53	0.74	0.76	0.57
Hungary	1110	0.55	345	0.52	0.49	0.79	0.8	0.55	1210	0.51	410	0.51	0.94	0.8	0.8	0.98
Ireland	917	0.51	278	0.52	0.74	0.74	0.71	0.41	**1630**	**0.49**	**380**	**0.55**	**0.03**	0.72	0.75	0.28
MKD	**1281**	**0.56**	**103**	**0.54**	**0.81**	**0.77**	**0.7**	**0.09**	**1529**	**0.54**	**104**	**0.59**	**0.34**	**0.77**	**0.86**	**0.04**
Poland	1797	0.59	371	0.62	0.23	0.81	0.82	0.65	1792	0.59	422	0.62	0.33	0.8	0.77	0.21
Romania	1158	0.71	83	0.66	0.38	0.77	0.8	0.53	1354	0.68	139	0.7	0.62	0.79	0.77	0.58
Scotland	1824	0.66	454	0.68	0.42	0.81	0.81	0.96	2036	0.59	401	0.56	0.22	0.77	0.78	0.68
Sweden	**2227**	**0.64**	**691**	**0.70**	**<0.01**	0.81	0.81	0.81	**2353**	**0.61**	**786**	**0.66**	**0.03**	0.81	0.83	0.29
Slovakia	1648	0.65	360	0.67	0.4	nd	nd	nd	1830	0.64	463	0.65	0.56	nd	nd	nd
Wales	1492	0.69	359	0.71	0.54	0.81	0.81	0.9	1591	0.64	320	0.62	0.61	0.78	0.75	0.22

LTID, Long-term illnesses or Disabilities; >2 h, two hours or more of TV viewing; *p*, *p*-value from Chi-square test of independence; MKD, former Yugoslav Republic of Macedonia; nd, no data. Bold text indicates *p* < 0.05.

**Table 2:** Proportion (0–1) of boys who spend two hours or more per day on computers during the weekdays and the weekend.

	Computer Games Weekdays			Computer Games Weekend			Use of Computer Weekdays			Use of Computer Weekends		
	No LTID	LTID		No LTID	LTID		No LTID	LTID		No LTID	LTID	
Country	>2 h	>2 h	*p*	>2 h	>2 h	*p*	>2 h	>2 h	*p*	>2 h	>2 h	*p*
All	**0.55**	**0.58**	**<0.01**	**0.73**	**0.74**	**0.01**	**0.52**	**0.54**	**0.01**	0.61	0.63	0.16
Armenia	0.37	0.33	0.57	0.53	0.49	0.53	0.4	0.45	0.35	0.55	0.55	0.96
Bulgaria	0.66	0.61	0.15	0.73	0.74	0.77	0.61	0.64	0.39	0.64	0.7	0.11
Czechia	0.6	0.63	0.19	0.7	0.7	0.90	0.52	0.52	0.96	0.57	0.56	0.54
England	0.53	0.54	0.72	0.73	0.73	0.73	0.53	0.56	0.24	0.65	0.69	0.11
Finland	**0.51**	**0.59**	**0.01**	0.71	0.73	0.49	0.48	0.47	0.72	0.57	0.56	0.79
France	0.48	0.49	0.66	0.75	0.71	0.07	0.46	0.46	0.85	0.59	0.57	0.54
Hungary	0.51	0.51	0.95	**0.75**	**0.82**	**0.02**	0.37	0.41	0.21	**0.53**	**0.6**	**0.03**
Ireland	0.37	0.4	0.29	0.61	0.61	0.96	0.43	0.46	0.43	0.56	0.56	0.95
MKD	0.47	0.53	0.27	0.7	0.72	0.73	0.51	0.54	0.48	0.68	0.65	0.55
Poland	0.48	0.48	0.85	**0.7**	**0.75**	**0.03**	**0.49**	**0.57**	**0.01**	**0.62**	**0.7**	**<0.01**
Romania	0.61	0.61	0.95	0.73	0.68	0.35	0.48	0.47	0.88	0.57	0.57	0.88
Scotland	0.63	0.67	0.20	0.78	0.8	0.30	0.58	0.6	0.35	0.67	0.67	0.90
Sweden	**0.62**	**0.68**	**0.01**	0.8	0.81	0.45	0.57	0.6	0.28	0.64	0.64	0.99
Slovakia	0.59	0.64	0.13	nd	nd	nd	0.53	0.55	0.58	nd	nd	nd
Wales	0.64	0.67	0.27	**0.76**	**0.81**	**0.05**	0.6	0.63	0.27	0.66	0.71	0.11

LTID, Long-term illnesses or Disabilities; >2 h, two hours or more of computing; *p*, *p*-value from Chi-square test of independence; MKD, former Yugoslav Republic of Macedonia; nd, no data. Bold text indicates *p* < 0.05.

**Table 3:** Proportion (0–1) of girls who spend two hours or more per day on computers during the week and the weekend.

	Computer Games Weekdays			Computer Games Weekend			Use of Computer Weekdays			Use of Computer Weekends		
	No LTID	LTID		No LTID	LTID		No LTID	LTID		No LTID	LTID	
Country	>2 h	>2 h	*p*	>2 h	>2 h	*p*	>2 h	>2 h	*p*	>2 h	>2 h	*p*
All	0.31	0.31	0.85	0.44	0.43	0.17	**0.53**	**0.57**	**<0.01**	**0.63**	**0.65**	**<0.01**
Armenia	0.22	0.3	0.13	0.38	0.38	0.93	**0.35**	**0.46**	**0.01**	0.51	0.72	0.64
Bulgaria	0.47	0.46	0.75	0.52	0.54	0.60	0.62	0.65	0.70	0.66	0.68	0.96
Czechia	0.25	0.22	0.27	0.32	0.32	0.83	0.5	0.52	0.14	0.55	0.6	0.46
England	**0.37**	**0.42**	**0.03**	**0.49**	**0.56**	**0.01**	**0.56**	**0.63**	**<0.01**	**0.67**	**0.58**	**<0.01**
Finland	0.08	0.09	0.75	0.14	0.14	0.75	**0.56**	**0.6**	**<0.01**	0.66	0.6	0.10
France	0.28	0.29	0.67	0.42	0.45	0.24	0.47	0.48	0.37	0.6	0.72	0.51
Hungary	0.31	0.28	0.26	0.52	0.48	0.22	0.4	0.43	0.38	0.56	0.72	0.41
Ireland	0.3	0.31	0.90	0.44	0.41	0.33	0.48	0.5	0.23	0.6	0.63	0.73
MKD	**0.25**	**0.36**	**0.02**	0.49	0.56	0.18	**0.49**	**0.58**	**<0.01**	**0.67**	**0.69**	**0.01**
Poland	0.2	0.23	0.24	0.36	0.36	0.83	0.54	0.6	0.21	0.66	0.67	0.09
Romania	0.35	0.39	0.31	0.46	0.48	0.75	**0.49**	**0.55**	**0.03**	0.61	0.63	0.67
Scotland	0.44	0.45	0.61	0.56	0.58	0.50	0.62	0.62	0.28	0.71	0.69	0.17
Sweden	**0.29**	**0.34**	**0.01**	**0.41**	**0.46**	**0.01**	**0.59**	**0.63**	**0.04**	**0.65**	**0.67**	**0.01**
Slovakia	0.31	0.35	0.06	nd	nd	nd	0.54	0.55	0.59	nd	nd	nd
Wales	0.39	0.35	0.18	0.52	0.51	0.77	0.6	0.62	0.93	0.68	0.7	0.56

LTID, Long-term illnesses or Disabilities; >2 h, two hours or more of computing; *p*, *p*-value from Chi-square test of independence; MKD, former Yugoslav Republic of Macedonia; nd, no data. Bold text indicate *p* < 0.05.

## Appendix A

**Table A1:** Differences in Gender Means (hours per day) and Standard deviation of sum of screen time during the week and the weekend by countries.

	Total Screen Time on Weekdays					Total Screen Time on Weekends				
	Boys		Girls		*t*-Test	Boys		Girls		*t*-Test
Country	Mean	SD	Mean	SD	*p*	Mean	SD	Mean	SD	*p*
All	6.84	4.80	6.00	4.39	<0.001	9.35	5.50	8.08	5.09	<0.001
Armenia	5.39	3.92	4.57	3.59	<0.001	7.35	4.83	6.51	4.44	<0.001
Bulgaria	8.07	5.40	7.68	4.90	0.016	9.44	5.85	9.06	5.46	0.028
Czechia	6.93	4.53	5.30	3.80	<0.001	8.88	5.37	6.66	4.64	<0.001
England	6.63	4.68	6.59	4.74	0.806	9.39	5.35	8.86	5.23	0.002
Finland	6.11	3.72	5.00	3.01	<0.001	8.50	4.41	6.68	3.62	<0.001
France	6.37	5.06	5.63	4.66	<0.001	9.49	5.67	8.04	5.40	<0.001
Hungary	5.71	4.20	5.11	4.04	<0.001	9.38	5.35	8.16	5.13	<0.001
Ireland	5.19	4.07	5.47	4.47	0.066	7.70	5.13	7.63	5.15	0.716
MKD	6.32	4.93	5.60	4.60	<0.001	9.45	5.77	8.49	5.25	<0.001
Poland	6.26	4.58	5.48	3.84	<0.001	9.18	5.32	7.88	4.73	<0.001
Romania	7.25	4.98	6.35	4.45	<0.001	8.88	5.65	8.03	4.96	<0.001
Scotland	7.69	5.05	7.16	4.96	<0.001	10.19	5.55	9.41	5.46	<0.001
Sweden	7.54	4.79	6.31	4.11	<0.001	10.29	5.57	8.13	4.82	<0.001
Slovakia	7.10	4.85	5.97	4.20	<0.001	nd		nd		nd
Wales	7.74	4.82	6.73	4.58	<0.001	10.22	5.57	8.86	5.32	<0.001

MKD, former Yugoslav Republic of Macedonia; nd, no data.
